# Design considerations for an automated hydride evolution system based on continuous flow principles

**DOI:** 10.1155/S1463924680000405

**Published:** 1980

**Authors:** A. L. Dennis, D. G. Porter

**Affiliations:** Automation and Computing Section Laboatory of the Government Chemist Cornwall House Stamford Street London SE1 9NQ UK


### Design considerations for an automated hydride evolution system based on continuous flow principles

A.L. Dennis and D.G. Porter

*Automation and Computing Section, Laboatory of the Government Chemist, Cornwall House, Stamford Street, London SE1 9NQ, UK.*

### Introduction

Environmental concentrations of elements such as arsenic, selenium and, to a lesser extent, antimony, are of considerable interest because of their potential toxicity. The Laboratory of the Government Chemist is called on to monitor levels of arsenic and selenium in water and very sensitive methods for their measurement are therefore required.

Investigations have shown that adequate sensitivity can be obtained by using a modification of an automated hydride evolution procedure developed by Goulden and Brooksbank [1]. This procedure utilises continuous flow techniques to generate the hydrides which are then fed to an on-line atomic absorption spectrometer.

One of the inherent advantages of automation, compared with a manual approach, is that more precise control can be maintained in a routine environment. A very rigid control of both gas and liquid flows is essential for the successful operation of a continuous flow hydride evolution system, and this paper will describe the development of an automated system that has worked routinely in the authors' laboratory for several years, and which forms the basis of a commercially available system. The construction is modular and where possible readily available commercial components have been used. Particular attention has been paid to the safe and consistent pumping of corrosive materials and of slurries. The performance and potential for further development of the system will be discussed.

### Preliminary investigations

### Apparatus

The automated equipment consisted of a Technicon Sampler II, a Technicon Proportioning Pump, a Technicon Heating Bath containing a 10 ft and a 20 ft coil each of 2.4 mm ID glass tubing together with the stripping column, wash column and tube furnace used by Goulden and Brooksbank. The spectrometer used was an Instrumentation Laboratory 251 atomic absorption spectrometer equipped with hollow cathode lamps and chart recorder.

### Procedure

The manifold used in preliminary investigations (Figure 1) is similar to that described by Goulden and Brooksbank [1].

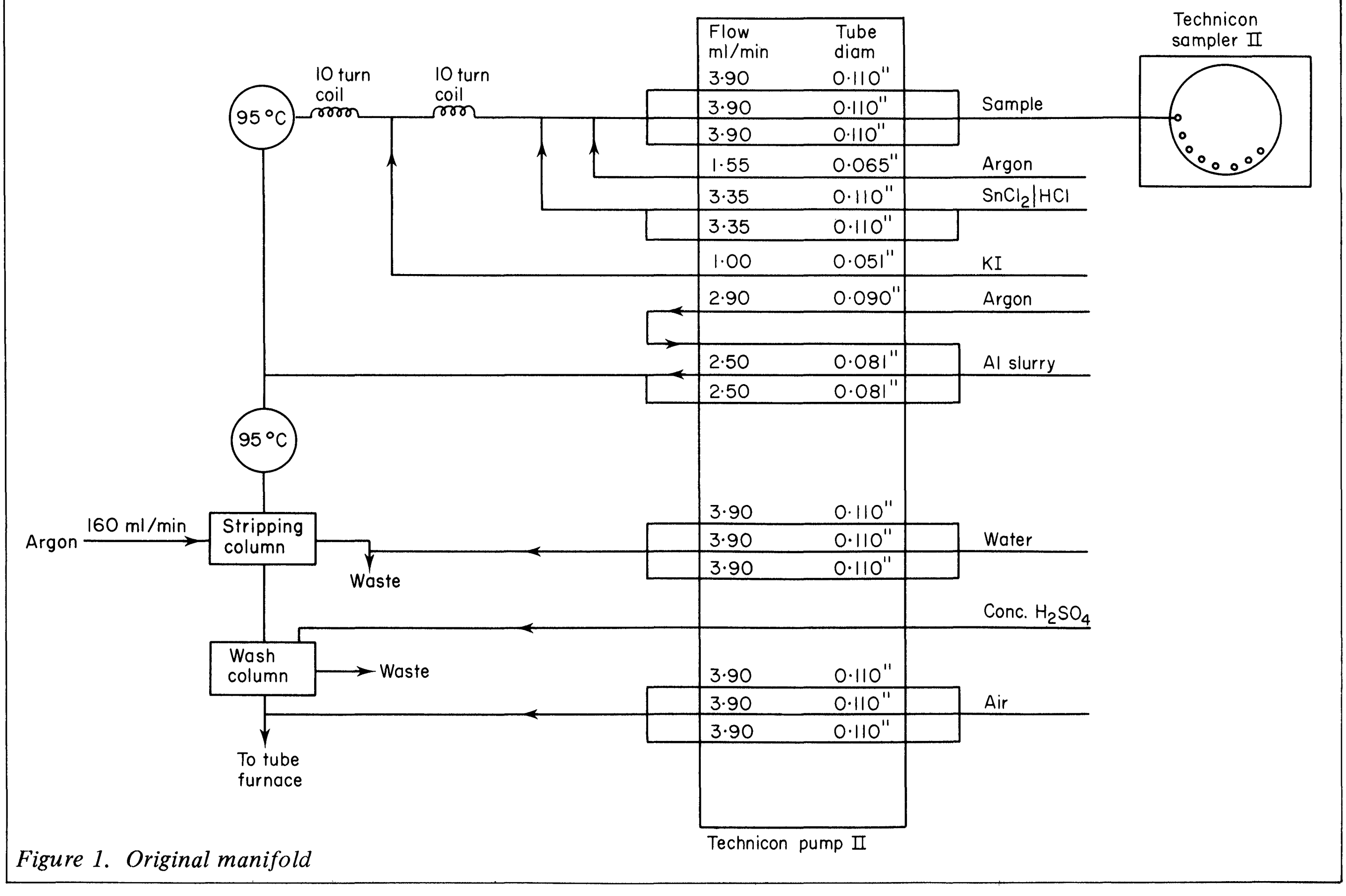

*Figure 1. Original manifold*

The sample is mixed with concentrated hydrochloric acid, stannous chloride, and potassium iodide and passed through the first coil of the 95°C heating bath. In this coil, any arsenate and selenate present in the sample are reduced to arsenite and selenite. The aluminium slurry is then added and the mixture is passed through the second coil of the heating bath. In this coil arsenite and selenite are further reduced to arsine and hydrogen selenide. The argon carrier gas is introduced and the gas-liquid mixture is carried to a stripping column where the gas and liquid phases are separated. The gas is cooled by passage through a water condenser, washed with concentrated sulphuric acid in a wash column, mixed with a small quantity of air and passed to a heated quartz tube which is fixed in the light beam of an atomic absorption spectrometer.

**Discussion**

*Pump*

The Technicon Pump II used was far from ideal, as in order to obtain the required flow rates, it was necessary to use three pump tubes in parallel on four streams. This was unsatisfactory, firstly, because such an arrangement can lead to very erratic flow patterns giving noisy signals at the detector thus reducing the precision of the analysis, and secondly, because of the cost of the pump tubes.

Furthermore, the Technicon Pump II is well known for the harsh treatment it metes out to pump tubes which consequently tend to have short lives, particularly when corrosive liquids such as concentrated sulphuric acid and concentrated hydrochloric acid are being pumped. Frequent renewal of pump tubes is not only expensive, it is also time consuming.

*Sampler*

The use of a Technicon Sampler II was adequate for the job in all but one respect. It would be useful to be able to use slightly larger sample cups than the Technicon unit will accommodate. When sampling at almost 12 ml/min there is no margin for error if a cup is even slightly underfilled.

*Heating bath*

The bath used was a standard Technicon unit to which a larger wattage heater had been fitted to cope with the high rate of heat abstraction. The temperature variation was of the order of ±0.3°C. It was thought that this could be improved by use of a modified heating bath built in the authors' laboratory [2].

*Performance*

After much investigation to determine the optimum operating parameters, detection limits of 0.1 μg/l were obtained for both arsenic and selenium. The coefficient of variation at the 5 μg/l level was approximately 5%. A typical recorder trace for arsenic determination is shown in Figure 2.

At the completion of these preliminary investigations it was concluded that although the Goulden and Brooksbank system was capable of giving an adequate performance in the hands of a skilled and experienced operator, it was not yet suitable for routine use. In addition to the above it was expensive to maintain, in terms of both time and components, it was liable to perform erratically on occasions and it required a considerable amount of supervision. The precision of measurement in the region of the detection limit was barely adequate and pressure on analysts to improve detection limits is an ever-present factor. It was decided, therefore, that a new system should be constructed that would provide much improved control over flow rates and operating temperatures with the aim of improving performance at low concentrations. The opportunity would be taken to make the system safer (pumping concentrated hydrochloric and sulphuric acid is a hazardous procedure) and more convenient and labour saving in use.

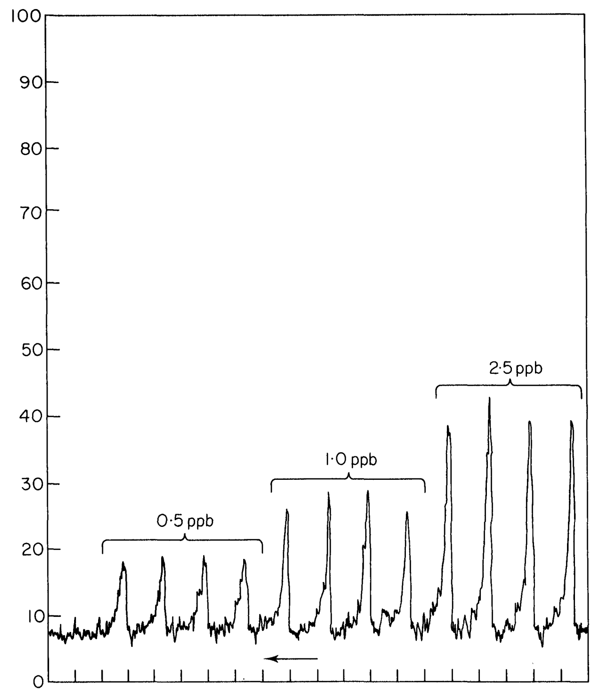

*Figure 2. Preliminary system – typical recorder trace of arsenic determination*

**Table 1. Pumping system (routine)**

| Reagents | Pump | RPM | Flow rate ml/min | Pump tube |
|---|---|---|---|---|
| Sample | 7016 | 15 | 12.0 | Tygon |
| Conc $H_2SO_4$ | 7014 | 15 | 3.0 | Viton |
| Argon (segmentation) | 7013 | 25 | 1.5 | Silicone |
| Al Slurry | 7014 | 25 | 5.1 | Tygon |
| $SnCl_2$/HCl | 7014 | 29 | 6.2 | Viton |
| KI | 7013 | 15 | 0.9 | Silicone |
| Argon (Al slurry) | 7014 | 15 | 3.1 | Silicone |
| Water | 7016 | 15 | 12.0 | Tygon |
| Air (oxidant) | – | – | 12.0 | – |
| Argon (carrier gas) | – | – | 220.0 | – |

### Development of routine system

**Apparatus**

The manifold now used routinely in the authors' laboratory is shown in Figure 3. The automated equipment consists of a modified Hook and Tucker A40 sampler, a Masterflex-based pumping system, a 95°C heating bath together with the stripping column, wash column and tube furnace used by Goulden and Brooksbank. The spectrometer used is a Perkin-Elmer 303 atomic absorption spectrometer equipped with electrodeless discharge lamps and chart recorder.

**Discussion**

*Pump*

The Masterflex-based pumping system (Table 1) consists of eight Masterflex pump heads mounted on four drive shafts, which are connected by toothed drive belts and pulley wheels to a single motor shaft which is driven by a high torque motor running at 26 rpm.

The use of this pumping system has led to a much smoother flow pattern and has therefore increased the precision of the analysis. The life of the pump tubes, which on the original system was less than one week, is now at least one month. This can be extended further by using longer lengths of tubing than are required and pulling the tubing through the pumps at regular intervals to expose a fresh length to the pump rollers. The expenditure on tubing has also been decreased, as only one tube is used for each reagent.

*Sampler*
The Hook the Tucker A40 sampler has been modified by the manufacturer to accommodate 20 ml sample cups which, together with the separate sample and wash timers, allows greater flexibility in operation to suit the particular analysis required.

*Heating Bath*
It has long been recognised by the authors that the standard Technicon heating bath has limitations in certain critical applications where close control of temperature is important. An improved heating bath has been constructed [2] that incorporates improved stirring, heat sensing and heat transfer. This bath will accommodate two standard Technicon 40 ft coils. Improved stirring is provided by a more powerful, faster stirring motor, the heating element is an unshielded nichrome wire coil in place of the more usual cartridge heater and the temperature sensor is a thermistor, which, having a very low mass, responds much more rapidly to temperature changes than does the bulb of the more usual mercury contact thermometer. This bath is controlled at 95°C ±0.02°C.

*Safety*
Because of the increasing emphasis on safety, particular care has been taken with the pumping of corrosive reagents. The stripping column and wash column are enclosed in a Perspex (transparent acrylic sheet) fronted cabinet which is designed to contain any spillages and protect the operator. It also protects the system from any draughts and thus improves control over the operating conditions.

*Construction*
The whole system is mounted on a trolley (100 cm long, 90 cm wide and 91 cm high) in order that it may be removed from the atomic absorption spectrometer when not in use, thus releasing an expensive analytical instrument for other use. The reagents are stored in a welded Cobex (rigid PVC sheet) lined compartment beneath the analytical system, which is sealed from the other lower compartments which contain the gearbox system and the power supplies for the stripping column, wash column and heating bath. Acid resistant grade Arborite (a high pressure thermoset plastic material consisting of multi-layers of specially processed papers impregnated with synthetic resins) has been used for all the shelves of the trolley as this has proved to be resistant to the reagents which are used. The lower compartments are enclosed by quick release panels to facilitate easy inspection.

**Table 2. As III for potable water**

| | n | Mean | sd | % recovery |
|---|---|---|---|---|
| Blank | 5 | 0.02 | 0.02 | – |
| 0.25ppb | 5 | 0.28 | 0.03 | – |
| 2.0ppb | 5 | 1.95 | 0.11 | – |
| Sample | 5 | 0.58 | 0.05 | – |
| Spiked sample | 5 | 1.59 | 0.02 | 107.37 |

*n = Number of observations*
*sd = Standard deviation*

*Performance*
The system has now been in routine use in the Water and Waste Water Sub-division of the authors' laboratory for about two years. It has proved to be reliable, economical and labour saving in operation. Its performance far exceeds that which can be obtained by manual methods. A typical recorder trace for arsenic determination is shown in Figure 4. The accuracy of the analytical results using the method laid down by the Standing Committee of Analysts of the Department of the Environment is shown in Table 2.

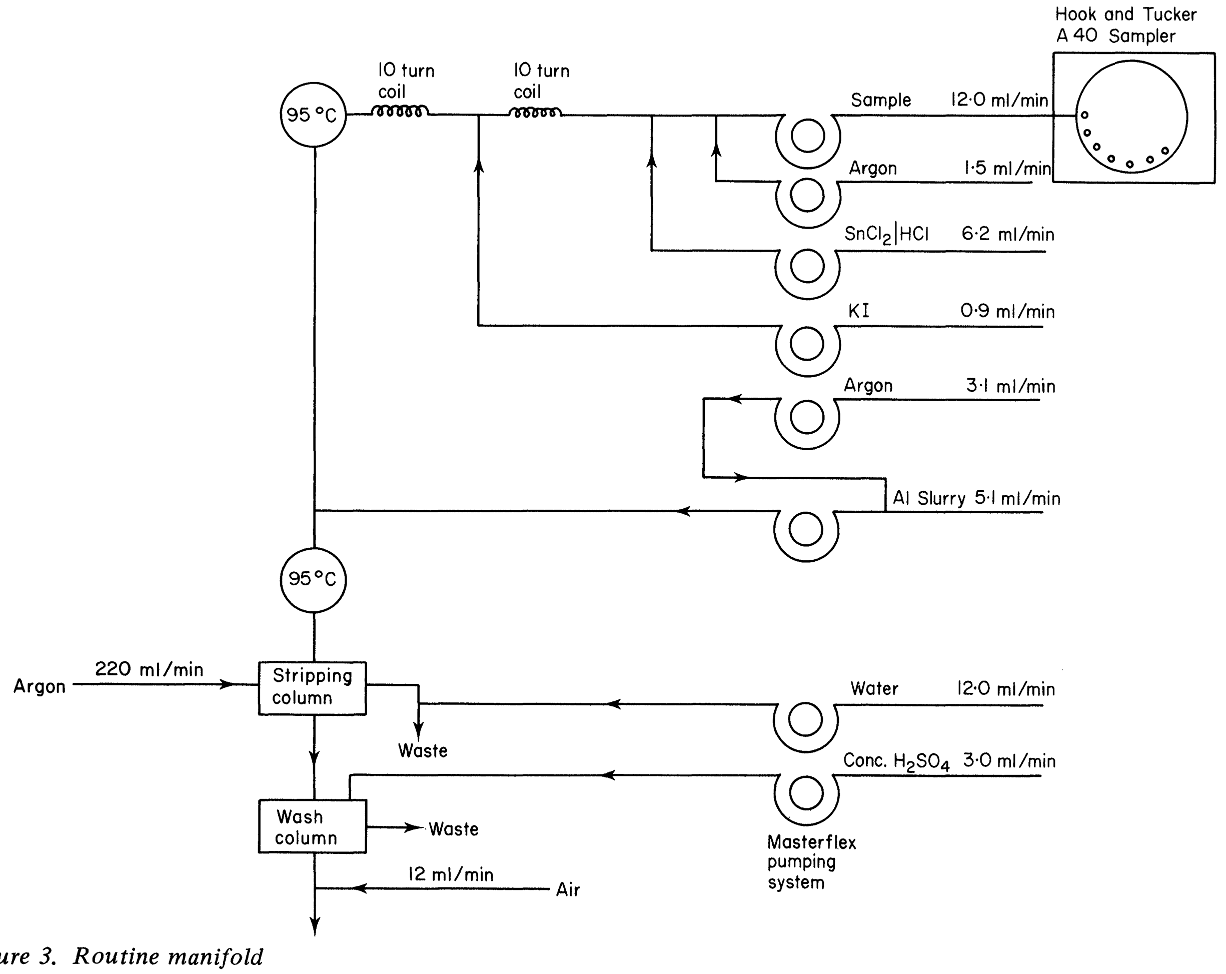

*Figure 3. Routine manifold*

### Small scale system

Since the completion of the routine system described above, investigations have proceeded on a smaller scale system in which reagent and gas flow rates are approximately one-half of those used in the routine system. It was hoped that the smoother flow patterns thus obtained would result in increased analytical performance.

Initial investigations used the standard stripping column and wash column of Goulden and Brooksbank, but it was soon apparent that in order to obtain the required analytical performance with these lower flow rates, it would be necessary to reduce the size of these columns. The glassware finally used, which is shown in Figure 5, has its linear dimensions reduced by about half.

The manifold used is the same as that for the routine system, except for the reagent and gas flows which are shown in Table 3.

The automated equipment consists of a modified Chemlab CS40 sampler, a Masterflex-based pumping system, a Chemlab high temperature bath and the tube furnace used by Goulden and Brooksbank. The spectrometer used is a Perkin-Elmer 403 atomic absorption spectrometer equipped with electrodeless discharge lamps and chart recorder.

### Discussion

*Pump*

The Masterflex-based pumping system is the same design as used in the routine system, except that different size pulley wheels are used connected to a motor running at 12.5 rpm.

*Sampler*

The Chemlab CS40 Sampler has been modified by the manufacturer to accommodate 20 ml sample cups, which allows the determination of both arsenic and selenium on the same sample aliquot.

*Heating bath*

The Chemlab high temperature bath has been modified by the addition of an extra 150 watt heating cartridge in order to cope with the high rate of heat abstraction. The temperature variation of this unit is ±0.1°C.

*Safety*

As in the routine system, the stripping column and wash column have been enclosed in a cabinet with a removable polycarbonate front. A Perspex cover has been fitted over the entire pumping system and between the heating bath outlets and the cabinet containing the stripping column and wash column.

All the reagents are stored in a Cobex lined compartment, beneath the analytical system, which is vented using a small air pump, the outlet of which is connected to the extract hood of the atomic absorption spectrometer.

All liquid wastes are fed to a 2 l container and then pumped to a suitable point using a PTFE pump (Chemlab Type PCM.150) designed to operate continuously.

*Construction*

The construction is identical to that of the routine system.

*Performance*

The detection limits (the concentrations which give signals twice that of the background noise) are 0.1 μg/l for both arsenic and selenium.

**Table 4. Statistics on peak height measurements.**

| | 1 μg/l | | | 2 μg/l | | | 10 μg/l | |
|---|---|---|---|---|---|---|---|---|
| | n | x | cv | n | x | cv | n | cv |
| As III | 9 | 40.8 | 2.8% | 11 | 82.6 | 2.9% | 11 | 1.9% |
| As IV | 11 | 34.4 | 5.3% | 11 | 67.7 | 1.0% | – | – |
| Se IV | 11 | 22.4 | 2.0% | 11 | 44.5 | 2.9% | 11 | 3.0% |
| Se VI | 11 | – | 2.1% | 11 | 49.0 | 2.6% | 11 | 2.6% |

*n = Number of observations*
*x = Mean peak height*
*cv = Coefficient of variation*

**Table 3. Pumping system (small scale)**

| Reagents | Pump | RPM | Flow rate ml/min | Pump tube |
|---|---|---|---|---|
| Sample | 7016 | 8.0 | 6.70 | Tygon |
| Conc $H_2SO_4$ | 7014 | 13.3 | 3.30 | Viton |
| Argon (segmentation) | 7013 | 13.3 | 0.53 | Silicone |
| Al slurry | 7014 | 16.6 | 3.70 | Tygon |
| $SnCl_2$/HCL | 7014 | 16.6 | 4.10 | Viton |
| KI | 7013 | 8.0 | 0.34 | Silicone |
| Argon (Al slurry) | 7014 | 8.0 | 2.00 | Silicone |
| Water | 7016 | 16.6 | 13.90 | Tygon |
| Air (oxidant) | – | – | 8.0 | – |
| Argon (carrier gas) | – | – | 80.0 | – |

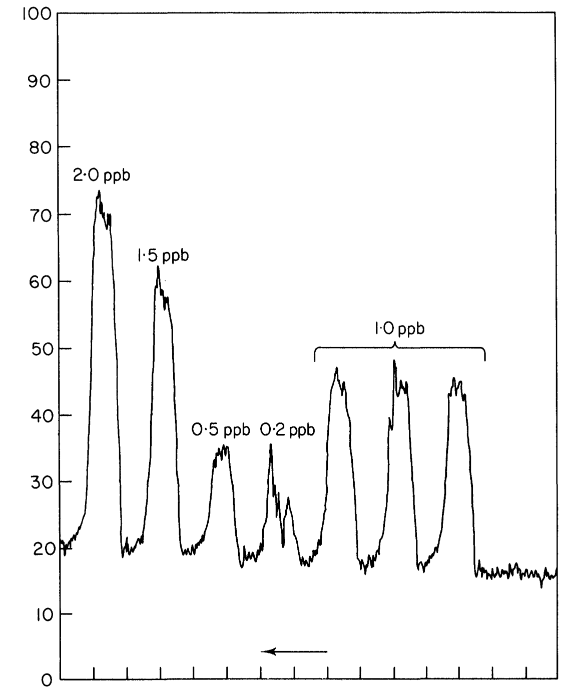

*Figure 4. Routine system – typical recorder trace of arsenic determination*

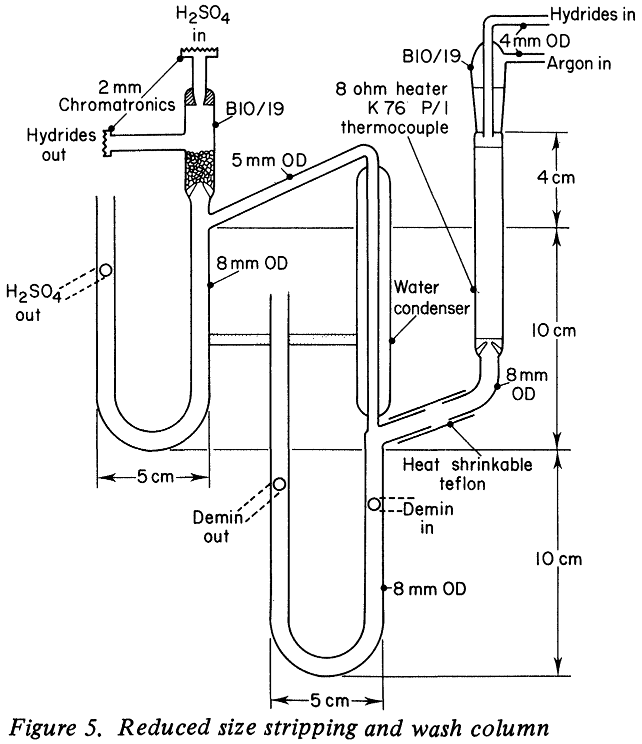

*Figure 5. Reduced size stripping and wash column*

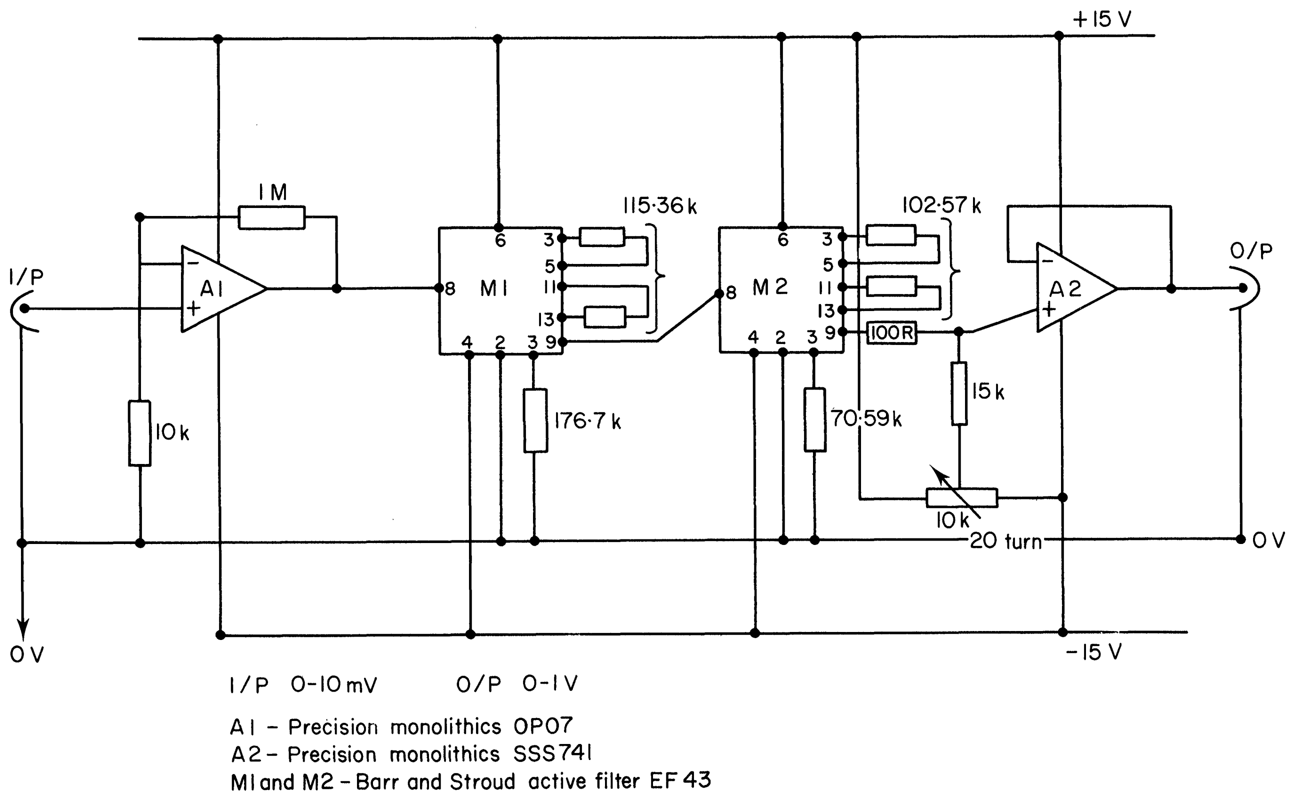

*Figure 6. Active filter – gain 100, 3 db cut-off, 0.03 Hz Bessel response*

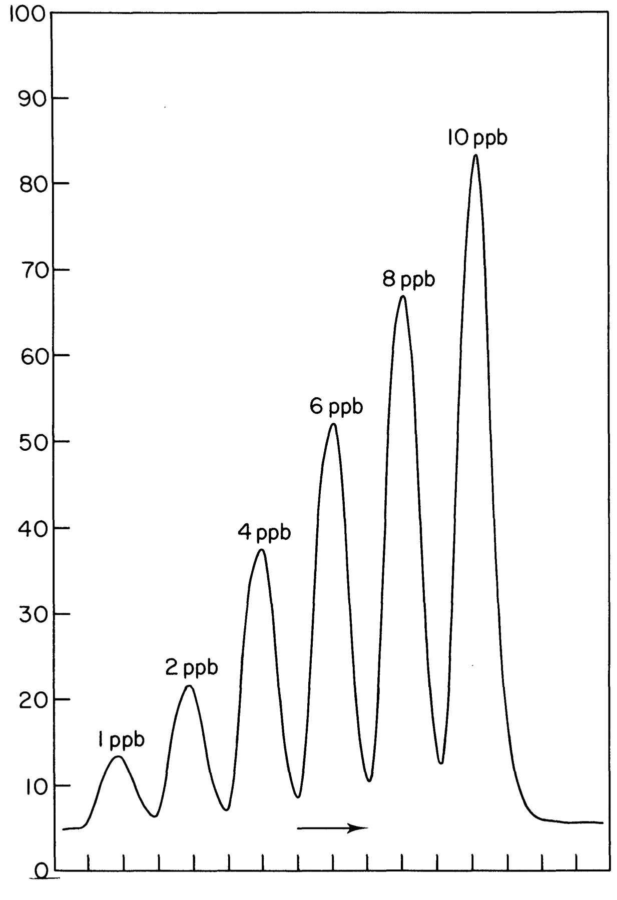

*Figure 7. Small scale system – typical recorder trace of arsenic determination*

The performance of this system has been further improved by the introduction of a low pass active filter (Figure 6) which has the effect of reducing unwanted noise from the signal without any degradation of the response due to the analyte. A typical recorder trace for arsenic determination using this filter is shown in Figure 7.

The coefficient of variation based on 11 replicate samples is shown in Table 4.

This system, built in the authors' laboratory, is currently in use at the Water Research Centre, Stevenage.

### Current development work

Currently work is being carried out on a system that links the hydride generation unit to an inductively-coupled plasma, operating with a microcomputer-controlled scanning monochromator. It is hoped to develop a system that will simultaneously determine arsenic, selenium, antimony and, possibly, bismuth and tellurium using sodium borohydride as reductant. This work will form the subject of further papers.

ACKNOWLEDGEMENTS

The authors would like to express their thanks to their colleagues, in several sections of the laboratory, who have contributed so much to the success of this work.

REFERENCES

[1] Goulden, P.D. and Brooksbank, P., *Analytical Chemistry,* 1974, **46,** 11, 1431-1436.

[2] Bunting, W., Lidzey, R.G., Porter, D.G. and Stockwell, P.B., *Laboratory Practice,* 1974, **23,** 179-190.

